# Clinical risk factors for Achilles tendinopathy: a systematic review

**DOI:** 10.1136/bjsports-2018-099991

**Published:** 2019-02-04

**Authors:** Arco C van der Vlist, Stephan J Breda, Edwin H G Oei, Jan A N Verhaar, Robert-Jan de Vos

**Affiliations:** 1 Department of Orthopedic Surgery, Erasmus MC University Medical Center, Rotterdam, The Netherlands; 2 Department of Radiology & Nuclear Medicine, Erasmus MC University Medical Center, Rotterdam, The Netherlands

**Keywords:** tendon, aetiology, causality

## Abstract

**Background:**

Achilles tendinopathy is a common problem, but its exact aetiology remains unclear.

**Objective:**

To evaluate the association between potential clinical risk factors and Achilles tendinopathy.

**Design:**

Systematic review.

**Data sources:**

The databases Embase, MEDLINE Ovid, Web of Science, Cochrane Library and Google Scholar were searched up to February 2018.

**Eligibility criteria:**

To answer our research question, cohort studies investigating risk factors for Achilles tendinopathy in humans were included. We restricted our search to potential clinical risk factors (imaging studies were excluded).

**Results:**

We included 10 cohort studies, all with a high risk of bias, from 5111 publications identified. There is limited evidence for nine risk factors: (1) prior lower limb tendinopathy or fracture, (2) use of ofloxacin (quinolone) antibiotics, (3) an increased time between heart transplantation and initiation of quinolone treatment for infectious disease, (4) moderate alcohol use, (5) training during cold weather, (6) decreased isokinetic plantar flexor strength, (7) abnormal gait pattern with decreased forward progression of propulsion, (8) more lateral foot roll-over at the forefoot flat phase and (9) creatinine clearance of <60 mL/min in heart transplant patients. Twenty-six other putative risk factors were not associated with Achilles tendinopathy, including being overweight, static foot posture and physical activity level.

**Conclusion:**

From an ocean of studies with high levels of bias, we extracted nine clinical risk factors that may increase a person’s risk of Achilles tendinopathy. Clinicians may consider ofloxacin use, alcohol consumption and a reduced plantar flexor strength as modifiable risk factors when treating patients with Achilles tendinopathy.

**Trial registration number:**

CRD42017053258.

## Introduction

The Achilles tendon is the largest and strongest tendon in the human body, yet it is prone to injury such as tendinopathy. The presence of Achilles tendon pain, swelling and an impaired load-bearing capacity indicate Achilles tendinopathy (AT).^1–3^ From a clinical perspective, insertional and midportion AT should be distinguished since these are two separate entities with different treatment approaches.^4^ AT is most frequently seen in elite running athletes, with a lifetime risk of 52%.^5^ It should, however, be noted that one-third of all patients with AT have a sedentary lifestyle.^6^ This emphasises that there is probably a broad spectrum of potential risk factors for AT, yet the exact aetiology remains uncertain.^7^

Over the last decades, various determinants have been proposed as risk factors in the development of AT. A recent systematic review examined risk factors for AT with a primary focus on genetic aspects.^8^ They found that certain genetic determinants may contribute to the development of AT, such as genetic contributors to collagen structure formation and tendon homeostasis. However, results were ambiguous due to the methodology in the publications included. These publications had mixed study designs, and the number of non-genetic clinical risk factors was limited. Therefore, there is a need to evaluate clinical risk factors in the development of AT with an extensive literature search and robust methodological design.

In this study, we systematically review the literature regarding the potential clinical risk factors that have been investigated for AT. This provides the level of evidence for all known clinical risk factors to inform future prevention and treatment strategies.

## Methods

### Protocol and registration

The protocol for this systematic review was prospectively registered in the international PROSPERO database. Protocol details can be accessed via http://www.crd.york.ac.uk/PROSPERO/display_record.asp?ID=CRD42017053258. A protocol revision was performed in July 2017 from midportion AT as site of injury to AT in general (insertional and midportion AT combined). This was done as we observed during data extraction that a substantial number of publications did not specify the specific location.

### Eligibility criteria

Publications were eligible for inclusion when there was: (1) a potential risk factor investigated in relation to AT and (2) a diagnosis of AT based on clinical findings (local pain and impaired load-bearing capacity). We restricted our selection to prospective and retrospective cohort studies written in English. A determinant was considered to be a potential clinical risk factor if no extensive examination (eg, biopsies) had been performed. Publications were excluded if there was: (A) no adequate control group (eg, contralateral Achilles tendon), (B) a preclinical study design or (C) an imaging study design (eg, potential risk factors derived from MRI or ultrasound examinations). Imaging studies were excluded, since these are regularly not directly available in the sports physician consultation room. For an overview of imaging, we refer to a recent systematic review.^9^

We also aimed to identify potential novel risk factors as secondary outcome measure by including cross-sectional studies. While the level of evidence from cross-sectional studies is lower than that from cohort studies, they might identify interesting factors to explore in future research.

### Literature search strategy and information sources

We conducted a sensitive search strategy for multiple databases with the assistance of a medical librarian (W M Bramer). The following databases were searched up to 12 February 2018: Embase, MEDLINE Ovid, Web of Science, Cochrane Library and Google Scholar. The search strategy is shown in online supplementary appendix 1.

10.1136/bjsports-2018-099991.supp1 Supplementary data

### Study selection and data extraction

Titles and abstracts were screened by two independent reviewers (ACvdV and RJdV) to identify eligible publications. Disagreements were solved by consensus, with the involvement of a third review author (EHGO) if necessary. Data extraction was performed by one author (ACvdV), and a data check was performed by a second author (SJB) for 100% of the primary outcomes and for 20% of the other data. This has been shown to be a methodologically sound procedure.^10^ The potential risk factors were extracted and grouped into patient characteristics (modifiable and non-modifiable), biomechanical factors, pre-existing diseases, medication and training factors. AT subgroup analysis results are presented in case subgroup analyses were performed in studies describing associations for multiple injuries. If multiple populations with AT were assessed in a single publication, only combined results are presented.

### Risk of bias assessment

Two reviewers (ACvdV and SJB) independently assessed the methodological quality of all included prospective (level of evidence II) and retrospective cohort studies (level of evidence III). No risk of bias assessment was performed for cross-sectional studies (level of evidence IV), since these studies are considered to be of high risk of bias for the purpose of this review.

To assess risk of bias, we used a standardised set of criteria based on modified questions of existing quality assessment tools (table 1).^11–13^ This tool has previously been used in a systematic review on risk factors for Achilles tendon rupture.^14^ If a criterion was met, one point was given. No points were given if the criterion was not met or when it was unclear if the specific criterion was met. A maximum score of 10 points could be obtained. Publications were considered to be of low risk of bias if: (1) a total score of 6 points or more was given and (2) 1 point was given to criteria 6, 7, 8 and 10.^14^

**Table 1:** Risk of bias assessment tool

Criteria	Response	Yes	No/not reported
**A clearly stated aim**	Did they have a ‘study question’ or ‘main aim’ or ‘objective’?	□	□
	The question addressed should be precise and relevant in light of available literature.	□	□
	To be scored adequate the aim of the study should be coherent with the ‘Introduction’ of the paper.	□	□
**Inclusion of consecutive patients**	Did the authors say: ‘consecutive patients’ or ‘all patients during period from … to….’ or ‘all patients fulfilling the inclusion criteria’?	□	□
**A description of inclusion and exclusion criteria**	Did the authors report the inclusion and exclusion criteria?	□	□
**Inclusion of patients**	Did the authors report how many eligible patients agreed to participate (ie, gave consent)?	□	□
**Prospective collection of data. Data were collected according to a protocol established before the beginning of the study**	Did they say ‘prospective’, ‘retrospective’ or ‘follow- up’? The study is not prospective when it is a chart review, database review, clinical guideline, or practical summaries.	□	□
**Outcome measures**	Did they report the association between the potential risk factors and manifestation of Achilles tendinopathy as outcome? The valid outcome measure for Achilles tendinopathy is clinical examination.	□	□
**Unbiased assessment of the study outcome and potential risk factors**	To be judged as adequate, the following two aspects had to be positive: Outcome and potential risk factors had to be measured independently. The outcome and potential risk factors for both cases and controls had to be assessed in the same way.	□	□
**Were the determinant measures used accurate (valid and reliable)?**	For studies where the determinant measures are shown to be valid and reliable, the question should be answered adequate. For studies that refer to other work that demonstrates the determinant measures are accurate, the question should be answered as adequate.	□	□
**Loss to follow-up**	To be judged as adequate, the following two aspects had to be positive: Did they report the losses to follow-up? Loss to follow-up was <20%.	□	□
**Adequate statistical analyses**	To be judged as adequate the following two aspects had to be positive: There must be a description of the relationship between the potential risk factors and Achilles tendinopathy (with information about the statistical significance). Was there adjustment for possible confounders (age, sex and body mass index) by multivariate analysis?	□	□

For each methodological criterion that is met 1 point is given. If the criterion was not met, zero points were given. Publications were considered to be of low risk of bias if: (1) a total score of at least 6 points was given and (2) 1 point was given to questions 6, 7, 8 and 10 (marked with the grey columns).

### Data synthesis

A subgroup analysis was initially planned for insertional and midportion AT; however, we revised the PROSPERO protocol (revision date 20 July 2017) because a substantial number of publications did not specify the AT location. Homogeneity of the data was evaluated, and if data could not be pooled because of heterogeneity, a best evidence synthesis based on the study of van Tulder *et al* was carried out for each potential risk factor.^15^

1. Strong evidence: ≥2 studies with high quality and generally consistent findings in all studies (≥75% of the studies reported consistent findings).
2. Moderate evidence: one high-quality study and ≥2 low-quality studies and generally consistent results (≥75% of the studies reported consistent findings).
3. Limited evidence: generally consistent findings in ≥1 low-quality study (≥75% of the studies reported consistent findings).
4. Conflicting evidence: <75% of the studies reporting consistent findings.
5. No evidence: no studies could be found.

## Results

### Study selection

**Figure 1:**
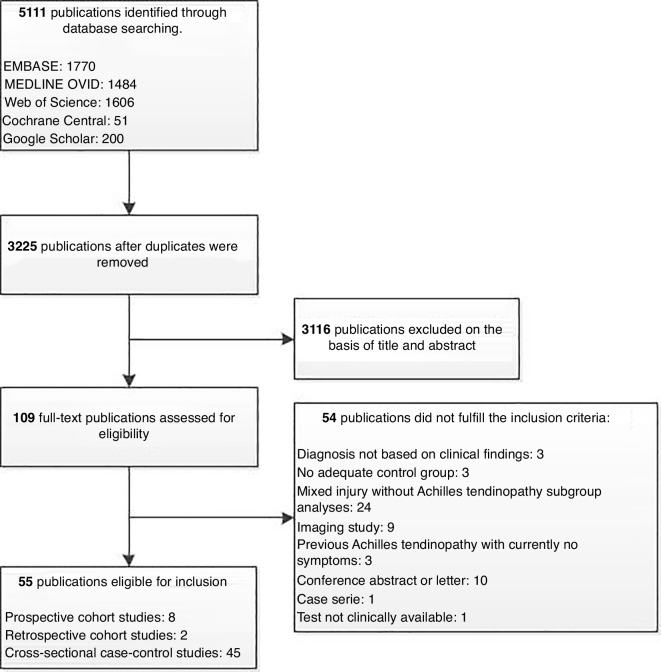
PRISMA 2009 flow diagram of study selection process. PRISMA, Preferred Reporting Items for Systematic Reviews and Meta-Analyses.

We identified 5111 potentially relevant publications, and after removing duplicates, 3225 remained. After screening title and abstract, we assessed 109 publications in full text. Fifty-four publications were excluded for different reasons after full-text evaluation, as shown in the Preferred Reporting Items for Systematic Reviews and Meta-Analyses (PRISMA) flow chart (figure 1). We included the remaining 55 publications for analysis.

### Characteristics of the included publications

We included eight prospective cohort studies^16–23^ and two retrospective cohort studies.^24 25^ Additionally, 45 cross-sectional studies were included.^5 26–69^ The characteristics and main findings of the included studies are summarised in table 2 for the cohort studies and online supplementary appendix 2 for the cross-sectional studies.

**Table 2:** Data extraction of the included prospective and retrospective cohort studies

Study	Study type	Duration of follow-up (weeks)	Participants (total group and cases of AT)	Sex (% male)	Age, mean±SD (years)	Location injury	Risk factors (risk ratio, OR and HR)	Quality score (points)
Barge-Caballero *et al* 24	RC	NR	149 (14); heart transplant patients who were prescribed quinolones.	77.9%	58.8±10.6	AT (not specified midportion or insertional).	A creatinine clearance <60 mL/min was associated with AT compared with a creatinine clearance ≥60 mL/min (OR 6.14; 95% CI 1.23 to 30.64; p=0.03). Increased time (in years) between heart transplantation and initiation of quinolone treatment for infectious disease was associated with AT (OR 1.39; 95% CI 1.11 to 1.74; p=0.005). No associations were found for age, sex, levofloxacin use and daily prednisone dose (mg).	5
Hein *et al* 20	PC	52	269 (10); recreational runners.	NR	NR	AT (not specified midportion or insertional).	No statistical analyses were performed.	4
Kaufman *et al* 21	PC	104	449 (30); Navy Sea, Air and Land (SEAL) candidates.	100%	22.5±2.5	AT (not specified midportion or insertional).	A tight ankle dorsiflexion with knee extended (<11.5°) was associated with AT compared with a normal dorsiflexion (11.5–15.0°) (RR 3.57; 95% CI 1.01 to 12.68; p<0.05). No associations were found for hindfoot inversion, hindfoot eversion, static arch index of the foot, dynamic arch index of the foot and dorsiflexion of the ankle with the knee bent.	5
Mahieu *et al* 16	PC	6	69 (10); officer cadets.	100.0%	18.4±1.3	Midportion AT.	Isokinetic plantar flexion strength at 30°/s was decreased in patients who developed AT for both the right and the left leg and at 120°/s for the right leg (p=0.042, p=0.036 and p=0.029, respectively). Plantar flexion strength was measured using the Cybex Norm dynamometer, which measures strength at constant velocity. No associations were found for weight, BMI, length, physical activity level, Achilles tendon stiffness, isokinetic plantar flexion strength at 120°/s for the left leg, explosive gastrocnemius-soleus muscle strength (standing broad jump test) and passive and active ankle joint range of motion outcomes.	4
Milgrom *et al* 22	PC	14	1405 (95); infantry recruits.	100.0%	18.7±7	Midportion AT.	An increase in AT was seen when training in the winter season compared with summer training (p=0.001). No differences were found in height, weight, BMI, external rotation of the hip, tibial intercondylar distance, arch type, physical fitness performance (2 km run and maximum number of chin-ups and sit-ups done) and shoe type.	4
Owens *et al* 17	PC	52	80 106 (450); military service members.	70.3%	NR	AT (not specified midportion or insertional).	Being overweight and obesity were associated with AT compared with underweight or normal weight (AOR 1.29, 95% CI 1.04 to 1.59 and AOR 1.59, 95% CI 1.16 to 2.17, respectively) A prior lower limb tendinopathy or fracture was associated with AT (AOR 3.87, 95% CI 3.16 to 4.75). Moderate alcohol use (7–13 units per week for men, 4–6 units per week for women) was associated with AT compared with no alcohol use (AOR 1.33, 95% CI 1.00 to 1.76). A birth year of 1980 and later was associated with a decreased risk for AT compared with a birth year before 1960 (AOR 0.62, 95% CI 0.38 to 1.00). No associations were found for sex, ethnicity, smoking status and heavy alcohol use (14+ units per week for men, 7+ units per week for women).	6
Rabin *et al* 18	PC	26	70 (5); military recruits.	100.0%	19.6±1.0	Midportion AT.	Every 1° increase in ankle dorsiflexion with the knee bent was associated with a decreased risk for AT (OR 0.77; 95% CI 0.59 to 0.94). No associations were found for BMI and lower extremity quality of movement.	7
Van Ginckel *et al* 19	PC	10	129 (10); novice runners.	14.7%	39±10	Midportion AT.	An increased total anterior displacement of the Y-component of the centre of force was associated with a decreased risk for AT (OR 0.919; 95% CI 0.859 to 0.984; p=0.015). A more medial directed force distribution underneath the forefoot at forefoot flat was associated with a decreased risk for AT (OR 0.000; 95% CI 0.000 to 0.158; p=0.016). No associations were found for age, height, weight, BMI or physical activity score.	6
Van der Linden *et al* 25	RC	NR	10 800 (8); patients using fluoroquinolones (index group) or amoxicillin, trimethoprim, cotrimoxazole or nitrofurantoin (reference group).	29.8%	46.3 (SD NR)	AT (not specified midportion or insertional).	The use of ofloxacin was associated with AT compared with the reference group (AOR 10.1; 95% CI 2.20 to 46.04). No associations were found for fluoroquinolones as a group, ciprofloxacin use and norfloxacin use compared with the reference group.	3
Wezenbeek *et al* 23	PC	104	300 (27); first-year students.	47%	18.0±0.8	Midportion AT.	Female sex was associated with AT (HR 2.82, 95% CI 1.16 to 6.87). Height and body weight were increased in patients with AT (p=0.028 and p=0.015). No association was found for a pronated foot posture. No differences were found for BMI, rating of perceived exertion, hours of sports participation and leg dominance.	7

AOR, adjusted OR; AT, Achilles tendinopathy; BMI, body mass index; NR, not reported; PC, prospective cohort study; RC, retrospective cohort study; RR, risk ratio.

Of the 10 cohort studies, five studies included only participants with midportion AT and five studies did not specify the AT location. Sample sizes of the included cohort studies ranged from 69 to 80 106 participants (median 285) with the number of AT cases ranging from 5 to 450 (median 18). Mean age ranged from 18 years old to 59 years old (median 21). This relatively young median age was caused by the profession of most of the populations investigated (military recruits in five studies, students in one study). There was a greater proportion of male participants in seven cohort studies, compared with two studies in which there was a greater proportion of female participants (median percentage males 78%). Data regarding the number of participants, mean age or sex was incomplete in two studies. The follow-up period for the prospective cohort studies ranged from 6 weeks to 2 years (median 39 weeks).

Of the 45 cross-sectional studies, 15 studies included only participants with midportion AT, one study included patients with insertional AT and 29 studies did not specify the AT location. Sample sizes ranged from 20 to 57 725 participants (median 201).

### Risk of bias assessment

All 10 cohort studies were considered to be of high risk of bias according to the predefined criteria (tables 1 and 3). Seven cohort studies scored six points or higher; however, they were lacking clinical examination as valid outcome (two studies), a valid and reliable determinant measure (four studies), an unbiased assessment of the study outcome (one study) and/or adequate statistical analyses (three studies). As a result, at best, a limited evidence for the association between the potential risk factor and tendinopathy could be detected. Results of the best evidence synthesis are presented in table 4.

**Table 3:** Risk of bias assessment scores of the 10 included cohort studies

Study	Criteria										Total score	Risk of bias
	1	2	3	4	5	6	7	8	9	10		
Barge-Caballero *et al* 24	1	1	1	0	0	0	0	1	0	1	5	High
Hein *et al* 20	1	0	1	1	1	0	0	0	0	0	4	High
Kaufman *et al* 21	1	1	0	1	1	1	1	0	0	0	5	High
Mahieu *et al* 16	0	1	0	1	1	1	1	0	0	1	6	High
Milgrom *et al* 22	0	0	0	1	1	1	0	0	0	0	3	High
Owens *et al* 17	1	1	0	1	1	0	1	1	0	0	6	High
Rabin *et al* 18	1	1	1	1	1	1	1	0	1	1	9	High
Van der Linden *et al* 25	1	1	1	0	1	0	1	1	0	0	6	High
Van Ginckel *et al* 19	1	0	1	1	1	1	1	0	0	1	7	High
Wezenbeek *et al* 23	1	1	1	0	1	1	0	1	0	1	7	High

Outcomes of the risk of bias assessment tool as presented in table 1. Publications were considered to be of low risk of bias if: (1) a total score of at least 6 points was given and (2) 1 point was given to questions 6, 7, 8 and 10 (marked with the grey columns).

**Table 4:** Potential risk factors investigated in the 10 cohort studies as potential risk factor for Achilles tendinopathy

Potential risk factors	Study (first author and reference number)	Best evidence synthesis
**Patient characteristics (non-modifiable)**		
Age	Barge-Caballero =^24^, Owens birth year >1980 ↓,^17^ Van Ginckel =^19^	Conflicting evidence
Sex	Barge-Caballero =^24^, Owens =^17^ Wezenbeek female ↑^23^	Conflicting evidence
Ethnicity	Owens =^17^	Limited evidence for no association
Height	Mahieu =,^16^ Milgrom =,^22^ Van Ginckel =,^19^ Wezenbeek ↑^23^	Limited evidence for no association
Prior lower limb tendinopathy or fracture	Owens ↑^17^	Limited evidence for positive association
**Patient characteristics (modifiable)**		
Body mass index	Owens BMI >25.0 ↑,^17^ Mahieu =,^16^ Milgrom =,^22^ Rabin =,^18^ Van Ginckel =,^19^ Wezenbeek =^23^	Limited evidence for no association
Body weight	Mahieu =,^18^ Milgrom =,^22^ Van Ginckel =,^23^ Wezenbeek ↑^23^	Limited evidence for no association
Alcohol use	Owens 7–13 units per week for men, 4–6 units per week for women ↑,^17^ Owens 14+ units per week for men, 7+ units per week for women =^17^	Limited evidence for positive association (moderate alcohol use)
Smoking	Owens =^17^	Limited evidence for no association
Physical activity level and performance	Mahieu physical activity level =,^16^ Van Ginckel physical activity level=,^19^ Milgrom physical activity performance (2 km run and maximum number of chin-ups and sit-ups) =,^22^ Wezenbeek =^23^	Limited evidence for no association
**Biomechanical factors**		
Shoe type	Milgrom =^22^	Limited evidence for no association
Leg dominance	Wezenbeek =^23^	Limited evidence for no association
Limited non-weight-bearing ankle dorsiflexion with knee extended	Kaufman <11.5° ↑,^21^ Mahieu =^16^	Conflicting evidence
Increased non-weight-bearing ankle dorsiflexion with the knee bent	Mahieu =,^16^ Rabin ↓,^18^ Kaufman =^21^	Conflicting evidence
Hindfoot inversion	Kaufman =^21^	Limited evidence for no association
Hindfoot eversion	Kaufman =^21^	Limited evidence for no association
Static arch index of the foot	Kaufman =,^21^ Milgrom =^22^	Limited evidence for no association
Dynamic arch index of the foot	Kaufman =^21^	Limited evidence for no association
Pronated foot posture	Wezenbeek =^23^	Limited evidence for no association
Increase in isokinetic plantar flexor strength at 30° (low velocity)	Mahieu ↓^16^	Limited evidence for protective association
Explosive gastrocnemius-soleus muscle strength	Mahieu =^16^	Limited evidence for no association
External rotation of the hip	Milgrom =^22^	Limited evidence for no association
Tibial intercondylar distance	Milgrom =^22^	Limited evidence for no association
lower extremity quality of movement test	Rabin =^18^	Limited evidence for no association
Increased total displacement of the Y-component of the centre of the centre of force	Van Ginckel ↓^19^	Limited evidence for protective association
Increased medial directed force distribution	Van Ginckel ↓^19^	Limited evidence for protective association
**Pre-existing diseases**		
Renal dysfunction (creatinine clearance <60 mL/min)	Barge-Caballero ↑^24^	Limited evidence for positive association
increased time between heart transplantation and initiation of quinolone treatment for infectious disease	Barge-Caballero ↑^24^	Limited evidence for positive association
**Medication**		
Fluoroquinolones as group	Van der Linden =^25^	Limited evidence for no association
Levofloxacin	Barge-Caballero =^24^	Limited evidence for no association
Ofloxacin	Van der Linden ↑^25^	Limited evidence for positive association
Ciprofloxacin	Van der Linden =^25^	Limited evidence for no association
Norfloxacin	Van der Linden =^25^	Limited evidence for no association
Daily prednisone dose	Barge-Caballero =^24^	Limited evidence for no association
**Training factors**		
Training in the winter season	Milgrom ↑^22^	Limited evidence for positive association

Associations found in this systematic review are marked with the grey columns.

=no association; ↑ positive association; ↓protective association.

### Risk factors

#### Patient characteristics (non-modifiable)

##### Age

There is conflicting evidence that age affects the risk for AT. One cohort study reported in 2013 that a birth year of 1980 or later is associated with a decreased risk for AT.^17^ Two cohort studies showed no association.^19 24^

##### Sex

There is conflicting evidence that sex affects the risk for AT. One cohort study reported that being female is associated with AT.^23^ No association was demonstrated in two cohort studies.^17 24^

##### Ethnicity

There is limited evidence that ethnicity does not affect the risk for AT. One cohort study reported no increased risk for white (non-Hispanic), black (non-Hispanic) or other ethnicity.^17^

##### Height

There is limited evidence that height does not affect the risk for AT. No association was found in three cohort studies.^16 19 22^ One cohort study reported an increased height in patients with AT.^23^

##### Prior lower limb tendinopathy or fracture

There is limited evidence that a prior lower limb tendinopathy or fracture increases the risk for AT. One cohort study reported that a prior lower limb tendinopathy (plantar fascia, Achilles or patellar) or fracture (regardless side of injury) is associated with AT.^17^

#### Patient characteristics (modifiable)

##### Body mass index (BMI) and body weight

There is limited evidence that BMI or body weight do not affect the risk for AT. No association was found in five cohort studies for BMI^16 18 19 22 23^ and in three cohort studies for body weight.^16 19 22^ One cohort study found that being overweight (BMI ≥25.0) and obesity (BMI ≥30.0) are associated with AT.^17^ Another cohort study found that body weight is increased in people who develop AT.^23^

##### Alcohol use

There is limited evidence that moderate alcohol use increases the risk for AT. Moderate alcohol use was defined as 7–13 units per week for men and 4–6 units per week for women. One cohort study reported that moderate alcohol use is associated with AT compared with no alcohol use. No association was found for light alcohol use or heavy alcohol use compared with no alcohol use.^17^

##### Smoking

There is limited evidence that smoking is not associated with AT based on one cohort study.^17^

##### Physical activity level, physical activity performance and hours of sports participation

There is limited evidence that physical activity level, physical activity performance or hours of sports participation do not affect the risk for AT. Two cohort studies found no association between the physical activity level measured with the Baecke questionnaire and AT.^16 19^ One cohort study found no association between the physical activity performance (2 km run and maximum number of chin-ups and sit-ups) and AT.^22^ Another cohort study found no differences in hours of sports participation between patients with AT and unaffected controls.^23^

#### Biomechanical factors

##### Shoe type

There is limited evidence that the type of shoes is not associated with AT. One cohort study found no difference in AT incidence between modified basketball shoes and standard lightweight infantry boots.^22^

##### Leg dominance

There is limited evidence that leg dominance is not associated with AT based on one cohort study.^23^

##### Static and dynamic properties of the foot

There is limited evidence that hindfoot inversion, hindfoot eversion, the static arch index of the foot, the dynamic arch index of the foot and a pronated foot posture do not increase the risk for AT. One cohort study reported that hindfoot inversion, hindfoot eversion, the static arch index of the foot and the dynamic arch index of the foot are not associated with AT.^21^ Another cohort study also found no association between the static arch index of the foot and AT.^22^ The third cohort study found no association between a pronated foot posture and AT.^23^

##### Static and dynamic properties of the ankle

There is conflicting evidence that a decreased non-weight-bearing ankle dorsiflexion is associated with AT. One cohort study found that a limited ankle dorsiflexion (<11.5°) with the knee extended is associated with AT compared with a normal ankle dorsiflexion (11–15°).^21^ Another cohort study evaluating ankle dorsiflexion with the knee extended did not show an association.^16^ One cohort study found that a one degree increase in ankle dorsiflexion with the knee bent is associated with a decreased risk for AT.^18^ Two cohort studies evaluating ankle dorsiflexion with the knee bent demonstrated no association.^16 21^

Limited evidence was found that an increased isokinetic plantar flexor strength at 30°/s (low velocity) is associated with a decreased risk for AT. No association between explosive gastrocnemius-soleus muscle strength (measured with the standing broad jump test) and AT was found in this study.^16^

##### Static and dynamic properties of the knee

There is limited evidence that the tibial intercondylar distance is not associated with AT based on one cohort study.^22^

##### Static and dynamic properties of the hip

There is limited evidence that the amount of external rotation of the hip is not associated with AT based on one cohort study.^22^

##### Gait analysis

There is limited evidence that an abnormal gait pattern with decreased forward progression of the propulsion and a more lateral foot roll-over at the forefoot flat phase are associated with AT. One cohort study reported a protective association per millimetre increase in total displacement of the Y-component of the centre of force. This cohort study also reported a decreased risk for AT if the mediolateral pressure distribution ratio underneath the forefoot at forefoot flat phase increased.^19^ No associations were found in another cohort study for the lower extremity quality of movement test.^18^

#### Pre-existing diseases

##### Renal dysfunction

There is limited evidence that a creatinine clearance <60 mL/min is associated with AT in heart transplant patients. One cohort study reported an increased risk to develop AT in this specific group compared with heart transplant patients with a creatinine clearance ≥60 mL/min.^24^

##### Heart diseases

There is limited evidence that an increased time (in years) between heart transplantation and initiation of quinolone treatment for infectious disease is associated with AT. One cohort study described this association.^24^ This outcome was solely investigated in heart transplant patients that all received quinolone treatment. Therefore, heart transplantation and quinolone treatment cannot be evaluated as individual risk factors in this cohort study.

#### Medication

##### Fluoroquinolones

There is limited evidence that the use of ofloxacin is associated with AT. One cohort study found an increased risk to develop AT when using ofloxacin compared with other antibiotic drugs (without fluoroquinolones).^25^ This cohort study found no associations for fluoroquinolones as a group, ciprofloxacin and norfloxacin. Another cohort study found no association between levofloxacin use and AT specifically in heart transplant patients compared with no use of levofloxacin.^24^

##### Corticosteroids

There is limited evidence that daily oral prednisone dose is not associated with AT based on one cohort study.^24^

#### Training factors

##### Training season

There is limited evidence that training during cold weather is associated with AT. One cohort study found that the incidence of AT increased during recruit winter training compared with summer training.^22^

#### Potential risk factors evaluated in cross-sectional studies

In the 45 cross-sectional studies, 296 risk factors were investigated. One hundred and fifteen associations were found, mostly consisting of biomechanical factors (56 associations) or genetic factors (30 associations). All data are presented in online supplementary appendix 2.

## Discussion

### Summary of main findings

This is the first high-quality systematic review of clinical risk factors for Achilles tendinopathy. We identified 10 cohort studies, all of which had a high risk of bias and 45 cross-sectional studies.

There is limited evidence for the following nine risk factors: (1) prior lower limb tendinopathy or fracture, (2) use of ofloxacin antibiotics, (3) increased time between heart transplantation and initiation of quinolone treatment for infectious disease, (4) moderate alcohol use, (5) training during cold weather, (6) decreased isokinetic plantar flexor strength, (7) abnormal gait pattern with decreased forward progression of propulsion, (8) more lateral foot-roll over at the forefoot flat phase and (9) a creatinine clearance of <60 mL/min in heart transplant patients.^16 17 19 22 24 25^

Although other potential risk factors such as body weight or BMI, static foot posture measurements and physical activity level are often said to be risk factors in clinical practice, there is currently no scientific evidence that they are associated with AT.^16–19 21–24 70^

### Clinical implications

Our systematic review indicates that for AT prevention and treatment, the advice to patients might include: (1) to reduce the use of alcohol to less than 7 units per week for men and less than 4 units for women, (2) to avoid the use of ofloxacin if alternatives are available and (3) to improve plantar flexor strength by performing strengthening exercises of the calf muscles.^16 17 25^

Whether these interventions will be effective is unknown. For example, calf muscle exercises seem a plausible preventive intervention as decreased plantar flexor strength is a risk factor. However, eccentric calf muscle exercises did not decrease AT incidence in soccer players in a randomised trial.^71^ Further research is needed before we can state whether the logical interventions work or not.

Abnormal gait pattern with decreased forward progression of the propulsion and a more lateral foot roll-over at the forefoot flat phase were found to be risk factors for AT in novice runners. Van Ginckel *et al* stated that more research is needed to confirm these findings, since this gait pattern with a decreased forward progression might be commonly used in well-trained athletes to improve gait economy.^19 72^ Since the gait pattern was determined barefoot, it is also not known whether these findings can be extrapolated to the running population, as running shoes might alter running gait.^19^ Biomechanical characteristics in AT are discussed in more detail in a recent systematic review.^73^

Previous research showed the relationship between BMI, body weight or waist circumference and tendon pathology.^74^ The hypothesis of this relationship is primarily based on the fact that the absolute tendon load is increased and that increased cytokine levels (Prostaglandin E2, tumor necrosis factor-α and Leukotriene B4) cause low-grade inflammation in obese individuals.^75 76^ In our systematic review, we were not able to find an association between being overweight and AT.^16–19 22 23^ It should be noted that more than half of the cohort studies that investigated BMI as a risk factor were in adolescent populations, in which being overweight is less common.^77^ Arnoczky and colleagues hypothesised based on an animal model that being underweight is associated with AT, as a consequence of a catabolic state causing a decreased collagen production.^78^ This could lead to a U-shaped relationship for the BMI and AT, making it less likely that an association be found.^79^ More cohort studies are needed in heterogeneous populations.

Another striking finding is the limited evidence for the absence of an association between physical activity level and AT. Inconclusive results regarding physical activity level were previously also demonstrated in patellar tendinopathy.^80^ In the majority of the scientific literature, tendinopathy is described as ‘overuse injury’. It could be that ‘overuse’ or ‘physical activity level’ is not measured accurately enough to detect associations. It could also be hypothesised that a sudden change in load is more important than the absolute load that is currently being measured in studies. To date, there is, however, no convincing evidence that AT is a result of overuse.

Moderate alcohol use and increased time between heart transplantation and initiation of quinolone treatment for infectious disease were potential risk factors for AT.^17 24^ It is hard to hypothesise why these determinants are risk factors for AT. They might be a confounding factor, with lifestyle factors that influence AT risk. Another reason might be that these findings have been detected by chance. When a high number of analyses are performed, the chance of statistical significance findings increases. Adequate statistical methods can prevent possible coincidental findings. Another potential risk factor that is lacking a clear explanation is training during cold weather.^22^ The direct cause-effect relationship is not known, as the results might be influenced by temperature or type of surface. This particular study did not report information on the temperature or training surface during the training period. These results are therefore difficult to extrapolate.

### Research implications

Our review showed that the majority of potential risk factors have only been investigated in cohort studies with a low number of cases (median 18 cases). Professor Roald Bahr and colleagues demonstrated that 20–50 cases are needed to detect moderate to strong associations and even 200 cases to detect small to moderate associations.^81^ Therefore, most studies in this review are likely to be underpowered to detect associations. Sample sizes in future studies should therefore be considered carefully. Future studies should also distinguish insertional from midportion AT, since these are two separate entities. It has been suggested that compression forces due to the bony prominence of the calcaneus play a role in the development of insertional AT, while this does not occur in midportion AT.^4^ Combining these entities, as occurred in most studies, is not ideal.

There are several interesting determinants found in the cross-sectional studies for future research. Use of oral contraceptives and hormone replacement therapy were more common in patients suffering from AT.^38^ Only one research group investigated these factors; therefore, more research is needed to confirm these findings. Regarding lipid profile, Dr Jamie Gaida and colleagues reported that triglyceride level, triglyceride/high-density lipoprotein cholesterol ratio and apolipoprotein B were elevated in patients with AT.^34^ Hypertension prevalence was found to be increased in females in the study by Holmes and Lin, suggesting a possible relationship between blood flow circulation to the Achilles tendon and AT.^38^ However, all of these factors should be studied in a longitudinal study design since it is not clear whether there is a cause–effect relationship.

Genetic profiling is also a major topic in AT. Since 2002, 16 cross-sectional studies have evaluated the presence of genetic variations in AT.^26 29 33 37 39 44–49 51–55^ These genetic variations are linked to collagen structure, tendon or matrix homeostasis, apoptosis or inflammation pathways.^8^ This type of research provides more information regarding the biological pathways of the disorder. Future therapy strategies could focus on targeting these pathways.

### Strengths and limitations

The strength of this systematic review is that we performed this structured analysis according to the PRISMA guidelines.^82^ Consequently, we were able to include 10 cohort studies, whereas a different systematic review on this topic only included one cohort study.^8^ That recent systematic review provided an excellent overview of all the literature considering genetic variants in AT, but important publications considering non-genetic risk factors in AT were missing. By including these cohort studies, our methods provide evidence that can be used directly in a clinical setting. We were also able to present an overview of topics on which future research should focus. Despite our robust research design, there are also methodological limitations. First, we only selected publications written in the English language. Second, we were not able to pool data because of the heterogeneity. The strength of the associations could therefore only be evaluated with a best evidence synthesis and not with meta-analysis. Third, we chose to use a standardised set of criteria based on modified questions of existing quality assessment tools. This was recommended by Hayden *et al*, since no validated tools are available for systematic reviews on risk factors.^83^ Our tool used strict criteria, primarily considering clinical examination as valid outcome measure (criterion 6). Four cohort studies did not meet this criterion of which three of these studies used International Statistical Classification of Diseases and Related Health Problems (ICD) codes/search terms.^17 24 25^ The fourth study examined only runners with serious symptoms.^20^ These approaches to case-finding creates a bias. Furthermore, if this criterion would not be taken into account, the studies would still be considered to be of high risk of bias based on the other criteria. Fourth, the median age of the included cohort studies was 21 years due to the profession of most of the populations investigated. This is a relatively young age, since the mean age to develop AT is 30–60 years, and AT is therefore expected to be less common in these studies.^69^

## Conclusion

There is a lack of high-quality prospective studies investigating risk factors for AT. We found limited evidence for nine determinants as risk factor for Achilles tendinopathy: a history of lower limb injury, season of training, calf muscle strength, gait analysis parameters, moderate alcohol use, fluoroquinolone antibiotic treatment and suboptimal renal function in a specific heart transplant population. Research funding agencies should prioritise research into modifiable determinants as these could prove useful for AT prevention and treatment. Quality studies will use valid clinical examination (focal Achilles tendon pain in relation to load with impaired load-bearing capacity) as outcomes, valid and reliable risk factor measurements and adequate statistical analysis in heterogeneous populations.

**What is already known**

- Achilles tendinopathy is considered to be an overuse injury. However, the exact aetiology remains unclear.
- The disorder is most frequently seen in runners and running sports in the age range from 30 years old to 60 years old.
- Being overweight, chronic diseases that affect tendon quality (diabetes, rheumatoid arthritis or hypercholesterolaemia), the use of fluoroquinolones or statins, a reduced plantar flexor strength and a reduced ankle dorsiflexion are generally considered to be risk factors for Achilles tendinopathy. To date, conclusive evidence is missing.

**What are the new findings**

- There is a lack of high-quality studies regarding risk factors for Achilles tendinopathy.
- Ten cohort studies were identified, all with a high risk of bias.
- There is limited evidence for nine determinants as risk factors for Achilles tendinopathy: (1) prior lower limb tendinopathy or fracture, (2) use of ofloxacin antibiotics, (3) increased time between heart transplantation and initiation of quinolone treatment for infectious disease, (4) moderate alcohol use, (5) training during cold weather, (6) decreased isokinetic plantar flexor strength, (7) an abnormal gait pattern with decreased forward progression of propulsion, (8) more lateral foot roll-over at the forefoot flat phase and (9) creatinine clearance of <60 mL/min in heart transplant patients.
