# Supplementary material for: Clinical risk factors for Achilles tendinopathy: a systematic review
Source: Br J Sports Med. 2019 Feb 4;53(21):1352–61. doi: 10.1136/bjsports-2018-099991 (PMC6837257; doi:10.1136/bjsports-2018-099991)
Supplement: Supplementary data [file bjsports-2018-099991supp001.docx]

**Appendix 1. Literature search strategy**

| Embase.com | 1770 | 1733 |
| --- | --- | --- |
| Medline Ovid | 1484 | 560 |
| Web of Science | 1606 | 844 |
| Cochrane | 51 | 1 |
| Google Scholar | 200 | 87 |
| **Total** | **5111** | **3225** |

**Embase.com**

('achilles tendinitis'/exp OR ((tendinitis/de OR pathology/de) AND 'achilles tendon'/de) OR (((achilles OR calcaneal) AND (tendinitis* OR tendinopath* OR tendinosis* OR tendonitis* OR tendon-patholog*))):ab,ti) AND ('risk factor'/exp OR risk/de OR 'disease predisposition'/de OR 'genetic predisposition'/exp OR 'heredity'/de OR 'genetic association'/de OR 'genetic background'/de OR heritability/de OR 'genetic variability'/de OR 'gait'/de OR 'joint mobility'/de OR 'body posture'/de OR 'hyperlipidemia'/de OR 'hypercholesterolemia'/exp OR 'cholesterol blood level'/de OR 'drug induced disease'/de OR 'adverse drug reaction'/de OR 'rheumatoid arthritis'/de OR 'sarcoidosis'/de OR 'gout'/de OR 'spondyloarthropathy'/de OR 'sport injury'/de OR 'biophysics'/de OR biomechanics/de OR 'etiology'/exp OR 'achilles tendinitis'/exp/dm_et OR interleukin/exp OR 'disease association'/de OR obesity/de OR 'body weight'/de OR 'weight change'/de OR 'weight change'/de OR 'body mass'/de OR mechanics/de OR Pliability/de OR (risk OR predisposit* OR susceptib* OR genetic* OR heritabil* OR inherit* OR gait OR (foot NEAR/6 (posture* OR dynamic* OR static*)) OR mobil* OR flexib* OR hypercholesterol* OR hyperlipid* OR ((cholesterol* OR lipid*) NEAR/6 (blood OR level*)) OR ((drug OR medicat* OR pharmac*) NEAR/6 (induc* OR adverse* OR reaction*)) OR ((induced OR associat* ) NEAR/6 (tendinitis* OR tendinopath* OR tendinosis* OR tendonitis* OR tendon-patholog*)) OR (rheumat* NEAR/3 arthrit*) OR sarcoidos* OR gout* OR spondyloarthropath* OR spondylarthropath* OR ((sport OR athlet*) NEAR/6 (injur* OR induc*)) OR overus* OR biomechanic* OR biophysics* OR etiolog* OR aetiolog* OR pathogenes* OR (tendon* NEAR/3 (characteristic* OR shape OR composition*)) OR mechanic* OR strain* OR stiff* OR interleukin* OR obes* OR overweight* OR ((body OR change* OR gain) NEAR/3 weight) OR 'body mass' OR bmi OR pliabil* OR (foot NEAR/3 position*)):ab,ti)

**Medline Ovid**

(((Tendinopathy/ OR Pathology/) AND "achilles tendon"/) OR *"achilles tendon"/pa* OR (((achilles OR calcaneal) AND (tendinitis* OR tendinopath* OR tendinosis* OR tendonitis* OR tendon-patholog*))).ab,ti.) AND ("risk factors"/ OR risk/ OR exp "Disease Susceptibility"/ OR "Genetics"/ OR "genetic background"/ OR Heredity/ OR "gait"/ OR "Pliability"/ OR "Posture"/ OR "Hyperlipidemias"/ OR exp "hypercholesterolemia"/ OR "cholesterol"/bl OR "Drug-Related Side Effects and Adverse Reactions"/ OR "Arthritis, Rheumatoid"/ OR "sarcoidosis"/ OR exp "gout"/ OR "Spondylarthropathies"/ OR "Athletic Injuries"/ OR "biophysics"/ OR "Causality"/ OR "Tendinopathy"/et OR exp interleukins/ OR exp obesity/ OR exp "body weight"/ OR exp "Body Weight Changes"/ OR "Body Mass Index"/ OR mechanics/ OR (risk OR predisposit* OR susceptib* OR genetic* OR heritabil* OR inherit* OR gait OR (foot ADJ6 (posture* OR dynamic* OR static*)) OR mobil* OR flexib* OR hypercholesterol* OR hyperlipid* OR ((cholesterol* OR lipid*) ADJ6 (blood OR level*)) OR ((drug OR medicat* OR pharmac*) ADJ6 (induc* OR adverse* OR reaction*)) OR ((induced OR associat* ) ADJ6 (tendinitis* OR tendinopath* OR tendinosis* OR tendonitis* OR tendon-patholog*)) OR (rheumat* ADJ3 arthrit*) OR sarcoidos* OR gout* OR spondyloarthropath* OR spondylarthropath* OR ((sport OR athlet*) ADJ6 (injur* OR induc*)) OR overus* OR biomechanic* OR biophysics* OR etiolog* OR aetiolog* OR pathogenes* OR (tendon* ADJ3 (characteristic* OR shape OR composition*)) OR mechanic* OR strain* OR stiff* OR interleukin* OR obes* OR overweight* OR ((body OR change* OR gain) ADJ3 weight) OR "body mass" OR bmi OR pliabil* OR (foot ADJ3 position*)).ab,ti.)

**Cochrane**

((((achilles OR calcaneal) AND (tendinitis* OR tendinopath* OR tendinosis* OR tendonitis* OR tendon-patholog*))):ab,ti) AND ((risk OR predisposit* OR susceptib* OR genetic* OR heritabil* OR inherit* OR gait OR (foot NEAR/6 (posture* OR dynamic* OR static*)) OR mobil* OR flexib* OR hypercholesterol* OR hyperlipid* OR ((cholesterol* OR lipid*) NEAR/6 (blood OR level*)) OR ((drug OR medicat* OR pharmac*) NEAR/6 (induc* OR adverse* OR reaction*)) OR ((induced OR associat* ) NEAR/6 (tendinitis* OR tendinopath* OR tendinosis* OR tendonitis* OR tendon-patholog*)) OR (rheumat* NEAR/3 arthrit*) OR sarcoidos* OR gout* OR spondyloarthropath* OR spondylarthropath* OR ((sport OR athlet*) NEAR/6 (injur* OR induc*)) OR overus* OR biomechanic* OR biophysics* OR etiolog* OR aetiolog* OR pathogenes* OR (tendon* NEAR/3 (characteristic* OR shape OR composition*)) OR mechanic* OR strain* OR stiff* OR interleukin* OR obes* OR overweight* OR ((body OR change* OR gain) NEAR/3 weight) OR 'body mass' OR bmi OR pliabil* OR (foot NEAR/3 position*)):ab,ti)

**Web of science**

TS=(((((achilles OR calcaneal) AND (tendinitis* OR tendinopath* OR tendinosis* OR tendonitis* OR tendon-patholog*)))) AND ((risk OR predisposit* OR susceptib* OR genetic* OR heritabil* OR inherit* OR gait OR (foot NEAR/5 (posture* OR dynamic* OR static*)) OR mobil* OR flexib* OR hypercholesterol* OR hyperlipid* OR ((cholesterol* OR lipid*) NEAR/5 (blood OR level*)) OR ((drug OR medicat* OR pharmac*) NEAR/5 (induc* OR adverse* OR reaction*)) OR ((induced OR associat* ) NEAR/5 (tendinitis* OR tendinopath* OR tendinosis* OR tendonitis* OR tendon-patholog*)) OR (rheumat* NEAR/2 arthrit*) OR sarcoidos* OR gout* OR spondyloarthropath* OR spondylarthropath* OR ((sport OR athlet*) NEAR/5 (injur* OR induc*)) OR overus* OR biomechanic* OR biophysics* OR etiolog* OR aetiolog* OR pathogenes* OR (tendon* NEAR/2 (characteristic* OR shape OR composition*)) OR mechanic* OR strain* OR stiff* OR interleukin* OR obes* OR overweight* OR ((body OR change* OR gain) NEAR/2 weight) OR "body mass" OR bmi OR pliabil* OR (foot NEAR/2 position*))))

**Google scholar**

"achilles|calcaneal tendinitis|tendinopathy|tendinosis" "risk factor|factors"|predisposition|predisposing|susceptibility|"disease association"|etiology|aetiology|etiologic|aetiologic

**Appendix 2. Data extraction of the included cross-sectional studies**

| **Study** (author and year) | **Participants**  (cases/controls) | **Sex (% male)** | **Age, mean ± SD (years)** | **Location injury** | **Main outcome** (Risk ratio, odd’s ratio, hazard ratio) |
| --- | --- | --- | --- | --- | --- |
| **Patient characteristics** | | | | | |
| De Jonge et al. (2011) (60) | 207/57 725; individuals registered in primary care | AT 47.7%, CON 48.1% | NR | Midportion Achilles tendinopathy | - No differences were found between sex and AT incidence. |
| Di Caprio et al. (2010) (58) | 40/68; recreational and competitive runners | NR | NR | Achilles tendinopathy (not specified midportion or insertional) | - Years of activity, weekly mileage (per kilometer), running on athletics tracks (compared to street or field surface) and flat arch of the foot were associated with AT (OR 1.13, 95% CI 1.06-1.21, p=0.001; OR 1.03, 95% CI 1.01-1.05, p=0.06, OR 5.25, 95% CI 1.26-21.84, p=0.023 and OR 16.99, 95% CI 2.76-104.40, p=0.002 respectively) - A valgus hindfoot was associated with AT (OR 0.32, 95% CI 0.12-0.86, p=0.024) - Days of practice per week and the use of spike shoes were higher in AT (p=0.002 and p=0.029 respectively) - No differences were found in age, sex, height, weight, BMI, running specialty, varus hindfoot and cavus arch |
| Gaida et al. (2016) (59) | 22/10; AT subjects and healthy controls | AT 60%, CON 68% | AT 53.0 ± 10.8, CON 54.5 ± 8.4 | Midportion Achilles tendinopathy | - Serum TNF-α and PDGF-BB levels were elevated in women with AT (p=0.014 and p=0.023 respectively) - No differences were found for serum IL-1β, bFGF, INF-ϒ and VEGF levels in both males and females and for TNF-α and PDGF-BB levels in males. |
| Gaida et al. (2009) (33) | 60/60; AT subjects and healthy controls | AT 53%, CON 53% | AT 47.9 ± 9.4, CON 46.6 ± 9.7 | Midportion Achilles tendinopathy | - Triglyceride level, triglyceride/ HDL-cholesterol ratio and apolipoprotein B were elevated in patients with AT (p=0.039; p=0.036 and p=0.017 respectively) - The percentage HDL-cholesterol was reduced in patients with AT (p=0.016) - No differences were found in total cholesterol level, LDL-cholesterol, LDL-cholesterol/HDL-cholesterol ratio, lipoprotein A, Apolipoprotein A1 and Apolipoprotein B/A1 ratio |
| Gouveia-Figueira et al. (2015) (34) | 15/16; AT subjects and healthy controls | AT 60.0%, CON 68.75% | AT 48, CON 47 | Midportion Achilles tendinopathy | - Oxylipin linoleic acid derivates 13-HODE, 12,13-DiHOME and 9,10,13-TriHOME were elevated in patients with AT (p=0.0012; p<0.0001 and p=0.0015 respectively) - No differences were found for all 21 arachidonic acid derivates and 6 other linoleic acid derivates |
| Holmes et al. (2006) (37) | 82/100; AT subjects and patients with other foot and ankle injuries as controls | AT 46.3%, CON NR | AT 50.5, CON NR | Achilles tendinopathy (not specified midportion or insertional) | - The prevalence of obesity, hypertension, oral contraceptive pill use and hormone replacement therapy was increased in females with AT (p<0.025; p<0.01; p<0.025 and p<0.01 respectively) - No differences were found in prevalence of obesity or hypertension in males and diabetes in males and females |
| Klein et al. (2013) (40) | 472/472; AT subjects and patients with other foot and ankle injuries as controls | AT 51.9%, CON 51.9% | AT 51.2 ± 13.5, CON 52.0 ± 14.3 | Achilles tendinopathy (not specified midportion or insertional) | - Overweight (BMI 25.0-29.9), class I obesity (BMI 30.0-34.9), class II obesity (BMI 35.0-39.9) and morbidly obesity (BMI ≥40.0) were associated with AT compared with a normal body weight (OR 2.60, 95% CI 1.88-3.61; OR 3.81, 95% CI 2.58-5.63; OR 3.77, 95% CI 2.25-6.34 and OR 6.56, 95% CI 3.18-13.55 respectively) |
| Klemp et al. (1993) (41) | 88 (48 adult familial hyperlipidaemia , 16 juvenile familial hyperlipidaemia and 24 mixed hyperlipidaemia) /88; controls patients with normal fasting lipid profiles | AT 47.7%, CON 47.7% | AT 37.5, CON NR | Achilles tendinopathy (not specified midportion or insertional) | - The prevalence of AT was elevated in patients with adult familial hyperlipidaemia and mixed hyperlipidaemia (p<0.001 and p=0.009 respectively) - No difference was found in the prevalence of AT in juvenile familial hyperlipidaemia |
| Knobloch et al. (2008) (61) | 291 in total (cases/controls NR); running athletes | 85.2% in total  (cases/controls NR) | 42 ± 9 years in total (cases/controls NR) | Achilles tendinopathy (not specified midportion or insertional for all risk factors) | - Running on sand (compared to asphalt, forest trails and mountains), the running discipline of 1500-3000m and 5 km (compared to marathon, half-marathon, 10 km, ultramarathon and triathlon) and >10 years of running experience were associated with AT (RR 10, 95% CI 1.12-92.8, p=0.011; RR 2.5, 95% CI 1.09-5.84, p=0.026; RR 1.8, 95% CI 1.04-3.18, p=0.034 and RR 1.6, 95% CI 1.02-2.76, p=0.041 respectively) - Running on asphalt (compared to sand, forest trails and mountains), performing the triathlon discipline and the half-marathon running discipline (compared to ultramarathon, marathon, 10 km, 5 km and 1500-3000m) were associated with AT (RR 0.47, 95% CI 0.25-0.89, p=0.019; RR 0.47, 95% CI 0.05-0.98, p=0.034 and RR 0.5, 95% CI 0.28-0.92, p=0.025 respectively) |
| Kraemer et al. (2012) (62) | 161/89; athletes | AT 68% and CON 64% | AT 41 ± 11, CON 39 ± 11 | Achilles tendinopathy (not specified midportion or insertional) | - A positive family history was associated with AT (OR 4.8, 95% CI 1.1-21.4, p=0.023) - Smoking and coronary artery disease were associated with AT (OR 0.2, 95% CI 0.1-0.4, p=0.001 and OR 0.0, p=0.001 respectively) - No differences were found in the prevalence of diabetes mellitus, hypercholesterolaemia or hypertension and no differences were found in the usage of corticosteroids, salicylate acids and consecutive therapy regimes for diabetes mellitus (dietetics, metformin, sulfonylureas or insulin), hypercholestolaemia (dietetics, fibrates or HMG-CoA-inhibitors) and arterial hypertension (dietetics, beta-adrenoceptor-blockers or ACE-inhibitors) |
| Kujala et al. (2005) (5) | 785/416; Former elite athletes and healthy military members as controls | AT 100%, CON 100% | AT 69.0, CON 67.5 | Achilles tendinopathy (not specified midportion or insertional) | - Middle and long-distance elite running (compared to other sports disciplines, e.g. soccer, ice hockey and boxing) and being an elite athlete were associated with AT (OR 31.2, 95% CI 13.5-71.8, p<0.001 and OR 5.21, 95% CI 3.20-8.49, p<0.001 respectively) |
| Plinsinga et al. (2018) (68) | 30/11; AT subjects and healthy controls | AT 56.7%, CON 54.5% | AT 45.7 ± 11.7, CON 41.0 ± 17.8 | Achilles tendinopathy (60% midportion Achilles tendinopathy, 27% insertional Achilles tendinopathy and 13% mixed symptoms) | - BMI was increased in AT (p=0.001) - No differences were found for total activity per week, thermal measures (cold detection threshold, warm detection threshold, thermal sensory limen, paradoxical heat sensation, cold pain threshold and heat pain threshold) and for mechanical measures (mechanical detection threshold, mechanical pain threshold, mechanical pain sensitivity, mechanical allodynia, wind-up ratio, vibration disappearance threshold and pain pressure threshold). |
| Scott et al. (2013) (63) | 197/100; AT subjects and patients with other foot and ankle injuries as controls (excluding plantar fasciopathy) | AT 57.3%, CON 38% | AT 52.77 ± 11.8, CON 42.74 ± 12.1 | Achilles tendinopathy (not specified midportion or insertional) | - BMI was increased in AT (p<0.001) |
| **Biomechanical factors** | | | | | |
| Azevedo et al. (2008) (26) | 21/21; long-distance runners | AT 76.19%, CON 76.19% | AT 41.8 ± 9.7, CON 38.9 ± 10.1 | Midportion Achilles tendinopathy | - Range of knee between heel strike and midstance, integrated electromyographic activity m. tibialis anterior 100 ms before heel strike and integrated electromyographic activity m. rectus femoris and m. gluteus medius 100 ms after heel strike were decreased in AT (p=0.011; p=0.003; p=0.000 and p=0.004 respectively) - No differences were found in hip angle at heel strike, hip angle at toe-off, hip range of motion, knee angle at initial supporting surface contact, knee angle in midstance, ankle angle at heel strike and ankle angle in midstance, integrated electromyographic activity 100 ms before and after heel strike for the m. peroneus longus, m. gastrocnemius lateralis and m. biceps femoris, integrated electromyographic activity 100 ms after heel strike for the m. tibialis anterior and integrated electromyographic activity 100 ms before heel strike m. rectus femoris and m. gluteus medius |
| Baur et al. (2011) (27) | 30/30; long-distance runners | AT 66.67%, CON 66.67% | AT 41 ± 7, CON 37 ± 10 | Midportion Achilles tendinopathy | - Neuromuscular activity of the m. peroneus longus and m. gastrocnemius medialis in weight acceptance phase and neuromuscular activity of the m. gastrocnemius medialis in the push-off phase were decreased in AT (p=0.006; p=0.001 and p=0.04 respectively) - No differences were found in neuromuscular activity of the m. tibialis anterior, m. peroneus longus and m. gastrocnemius medialis in preactivation, m. tibialis anterior during weight acceptance and m. tibialis anterior and m. peroneus longus in push-off phase |
| Becker et al. (2017) (66) | 13/13; long-distance runners | AT 69.2%, CON 69.2% | AT 37.6 ± 15.9, CON 32.6 ± 12.4 | Achilles tendinopathy (not specified midportion or insertional) | - Standing tibia varus angle and period of pronation were increased in AT (p<0.05 and p<0.05) - Dorsiflexion ROM and eversion at heel-off were decreased in AT (p<0.05 and p<0.05) - No differences were found for arch height index, plantarflexion ROM, hip internal rotation ROM, hip external rotation ROM, hamstring flexibility, quadriceps flexibility, subtalar inversion, subtalar eversion, first metatarsophalangeal joint ROM, peak eversion, excursion of eversion, time to peak eversion, time to heel-off, velocity of eversion, peak propulsive force, propulsive impulse and peak vertical force. |
| Child et al. (2010) (29) | 16/16; long-distance runners | AT 100%, CON 100% | AT 40 ± 8, CON 35 ± 9 | Midportion Achilles tendinopathy | - No difference was found in maximal isometric plantar flexion force |
| Chimenti et al. (2016) (65) | 20/20; AT subjects and healthy controls | AT 45%, CON 45% | AT 58.6 ± 7.8, CON 58.2 ± 8.5 | Insertional Achilles tendinopathy | - Plantar flexion during stair ascent and peak ankle plantar flexor strength was decreased in AT (p=0.017 and p=0.006 respectively) - End range dorsiflexion during stair ascent was increased in AT (p=0.026) - No differences were found in active dorsiflexion ROM, passive dorsiflexion ROM, isometric plantar flexion torque, peak dorsiflexion during stair ascent and peak ankle plantar flexor moment |
| Creaby et al. (2016) (30) | 14/11; long-distance runners | AT 100%, CON 100% | AT 43 ± 8, CON 37 ± 9 | Midportion Achilles tendinopathy | - Peak hip external rotation moment, hip external rotation impulse and hip abduction moment impulse were increased in AT (p=0.001; p<0.001 and p=0.001 respectively) - No differences were found in hip and ankle joint angles |
| Debenham et al. (2016) (31) | 15/11; AT subjects and healthy controls | AT 60.0%, CON 27.3% | AT 41.2 ± 12.7, CON 23.2 ± 6.7 | Midportion Achilles tendinopathy | - Lower limb stiffness, ankle angle 80 ms pre-contact, ankle angle at contact, peak ankle angle, soleus onset, tibialis anterior peak and tibialis anterior offset during hopping were increased in AT (p<0.001; p<0.05; p<0.05; p<0.05; p<0.001; p=0.026 and p<0.001 respectively) - No differences were found in soleus peak, soleus offset and tibialis anterior onset during hopping |
| Debenham et al. (2016) (67) | 13/13; AT subjects and healthy controls | AT 53.8%, CON 46.2% | AT 40.5 ± 8.9, CON 40.1 ± 15.6 | Midportion Achilles tendinopathy | - Two point discrimination (tactile acuity) was decreased in patients with AT (p=0.03) |
| Grigg et al. (2013) (35) | 11/9; AT subjects and healthy controls | AT 100%, CON 100% | AT 48.2 ± 8.5, CON 49.0 ± 10.3 | Midportion Achilles tendinopathy | - Peak dorsiflexion and ankle angular velocity during eccentric and concentric exercises were decreased in AT (p<0.05 respectively for both) - Peak plantar flexion during eccentric and concentric exercises was increased in AT (p<0.05) - The power spectrum during eccentric exercises shifted towards a lower frequency in AT (p<0.05) - No differences were found in ankle range of motion and power spectrum during concentric exercises |
| Kim et al. (2015) (39) | 20/20; recreational runners | AT 50%, CON 50% | AT 27.0 ± 4.6, CON 27.3 ± 4.3 | Achilles tendinopathy (not specified midportion or insertional) | - Double limb support, step width, stride time, hip joint moment during mid stance, terminal stance and pre-swing, knee joint moment during initial contact and ankle joint moment during pre-swing and terminal swing were increased in AT (p<0.05; p<0.05; p<0.05; p<0.01; p<0.001 p<0.01; p<0.01; p<0.05 and p<0.001 respectively) - Step length, stride length, walking speed, hip joint moment during initial contact and knee joint moment during pre-swing were decreased in AT (p<0.01; p<0.01; p<0.001; p<0.001 and p<0.01 respectively) - No differences were found in cadence, single-limb support, limb index, step time, hip joint moment during terminal swing, knee joint moment during mid stance, terminal stance and terminal swing and ankle joint moment during initial contact, mid stance and terminal stance |
| McCrory et al. (1999) (42) | 31/58; long-distance runners | NR | AT 38.4 ± 1.8, CON 34.5 ± 1.2 | Midportion Achilles tendinopathy | - Years of running experience, dorsiflexion peak torque/body weight ratio at 60 degrees/s and maximum pronation were increased in AT (p<0.05; p=0.05 and p=0.004 respectively) - Training pace, stretching habits, arch index, dorsiflexion peak torque at 60 degrees/s, plantar flexion peak torque at 180 degrees/s, time to maximum pronation and calcaneus to vertical touch down angle were decreased in AT (p<0.05; p<0.05; p<0.05; p=0.037; p=0.008; p=0.008 and p=0.017 respectively) - No differences were found in weekly mileage, competition pace, training surface, age, height, body mass, q-angle, ankle dorsiflexion range of motion, ankle plantar range of motion, plantar flexion park torque at 60 degrees/s, plantar flexion park torque/body weight ratio at 60 and 180 degrees/s, flexion/extension work ratio at 60 and 180 degrees/s, dorsiflexion peak torque at 180 degrees/s, dorsiflexion peak torque/body weight ratio at 180 degrees/s, plantar flexion total work at 180 degrees/s, plantar flexion endurance ratio at 180 degrees/s, plantar flexion average strength at 180 degrees/s, dorsiflexion total work at 180 degrees/s, dorsiflexion endurance ratio at 180 degrees/s, dorsiflexion average strength at 180 degrees/s, flexion/extension work ratio at 180 degrees/s, calcaneus to tibial touch down angle, pronation at 10% stance, total pronation range of motion, calcaneus to tibial toe off angle, eversion at 10% stance, maximum eversion, total eversion, time to maximum eversion, calcaneus to vertical toe off angle, initial velocity, maximum pronation velocity, time to maximum pronation velocity and all kinetic discriminators |
| Ryan et al. (2009) (49) | 27/21; long-distance runners | NR | AT 40 ± 7, CON 40 ± 9 | Midportion Achilles tendinopathy | - Subtalar joint eversion displacement during mid-stance of the running gait was increased in AT (p=0.04) - No differences were found in transverse plane tibial motion (internal/external rotation range of motion, internal rotation velocity, timing of maximum values of internal rotation velocity, external rotation velocity and timing of maximum values of external rotation velocity), sagittal plane ankle motion (dorsiflexion range of motion, plantarflexion range of motion, plantar flexion/dorsiflexion range of motion, plantar flexion velocity, timing of maximum values of plantarflexion velocity, dorsiflexion velocity and timing of maximum values of dorsiflexion velocity), frontal plane ankle motion (eversion range of motion, inversion range of motion, eversion/inversion range of motion, eversion velocity, timing of maximum values of eversion velocity, inversion velocity and timing of maximum values of inversion velocity), maximum values and timing of maximum values of internal tibial rotation, ankle dorsiflexion and ankle eversion |
| Smith et al. (2014) (55) | 14/19; long-distance runners | AT 100%, CON 100% | AT 43 ± 8, CON 37 ± 8 | Midportion Achilles tendinopathy | - A delayed activation of the m. gluteus medius and the m. gluteus maximus, a shorter duration of activation of the m. gluteus medius and the m. gluteus maximus and an earlier offset of muscle activity for the m. gluteus maximus was seen in AT (p<0.001; p=0.008; p<0.001; p=0.002 and p=0.001 respectively) - No differences were found in gluteus medius offset |
| Waldecker et al. (2012) (64) | 79/658; No or insignificant (1-4 hours per week) sports participation | AT 44.3%, CON NR | 50.3 years in total (cases/controls NR) | Achilles tendinopathy (not specified midportion or insertional) | - A varus alignment of the hindfoot was more frequent in AT (p<0.001) - The average tibiocalcaneal angle was decreased in AT (p<0.001) |
| Wyndow et al. (2013) (57) | 15/19; long-distance runners | AT 100%, CON 100% | AT 42 ± 7, CON 36 ± 8 | Midportion Achilles tendinopathy | - Relative offset timing between soleus and lateral gastrocnemius was earlier in AT (p=0.02) - No differences were found in onset timing between groups (lateral gastrocnemius and medial gastrocnemius, soleus and medial gastrocnemius and soleus and lateral gastrocnemius) and in offset timing between lateral gastrocnemius and medial gastrocnemius and soleus and medial gastrocnemius |
| **Genetic factors** | | | | | |
| Abrahams et al. (2013) (25) | 160/342; AT subjects and healthy controls | AT 73%, CON 50.6% | AT 39.8 ± 14.5, CON 37.7 ± 11.7 | Achilles tendinopathy (not specified midportion or insertional) | - COL5A1 rs71746744 AGGG/AGGG genotype (compared to -/AGGG and -/- genotypes), COL5A1 rs16399 -/- ATCT genotype (compared to ATCT/- and ATCT/ATCT genotypes), COL5A1 rs1134170 TT genotype (compared to AT and TT genotypes) and MIR608 rs4919510 CC genotype polymorphisms (compared to CG and GG genotypes) were independently associated with AT (OR 2.0; 95% CI 1.2-3.3; p=0.008, OR 1.7; 95% CI 1.1-2.7; p=0.015, OR 1.8; 95% CI 1.12-2.9; p=0.014 and OR 1.6; 95% CI 1.1-2.5; p=0.023) |
| Brown et al. (2016) (28) | 87/130; AT subjects and healthy controls | AT 58.6%, CON 63.1% | AT 45.2 ± 14.4, CON 41.6 ± 11.6 | Achilles tendinopathy (not specified midportion or insertional) | - Inferred I-G allele combination constructed from CASP8 rs3834129 and rs1045485 genotype (compared to D-G, D-C and I-C allele combination) was associated with AT (p=0.031) - CASP8 rs3834129 DD genotype (compared to DI, II and and I genotypes) was associated with AT (OR 0.45, 95% CI 0.22-0.90, p=0.020) - No associations were found in the genotype or allele frequency distributions for COL5A1 rs12722, COL5A1 rs3196378, COL5A1 rs71746744, MIR608 rs4919510, IL-1β rs16944, IL-6 rs 1800795 and CASP8 rs1045485. |
| El Khoury et al. (2016) (32) | 93/131; AT subjects and healthy controls | AT 58.1%, CON 62.6% | AT NR, CON 41.7 ± 11.6 | Achilles tendinopathy (not specified midportion or insertional) | - No associations were found in the genotype or allele frequency distributions for MMP3 rs679620 and TIMP2 rs4789932 |
| El Khoury et al. (2015) (38) | 135/239; AT subjects and healthy controls | AT 77.4%, CON 50.6% | AT 40.1 ± 14.2, CON 38.2 ± 11.2 | Achilles tendinopathy (not specified midportion or insertional) | - FBN rs331079 GG genotype (compared to GC and CC genotype) and FBN rs331079 G allele frequency (compared to C allele frequency) were associated with AT (OR 1.83, 95% CI 1.04-3.25, p=0.035 and OR 1.90, 95% CI 1.11-3.27, p=0.017 respectively) - No associations were found in the genotype or allele frequency distributions for ELN rs2071307 |
| Hay et al. (2013) (36) | 184/338; AT subjects and healthy controls | AT 69.4%, CON 51.5% | AT 40.8 ± 14.7, CON 38.0 ± 11.6 | Achilles tendinopathy (not specified midportion or insertional) | - TCT pseudohaplotype constructed from COL11A1 rs3753841, COL11A1 rs1676486 and COL11A2 rs1799907 and TCT (AGGG) pseudohaplotype constructed from COL11A1 rs3753841, COL11A1 rs1676486, COL11A2 rs1799907 and COL5A1 rs71746744 were overrepresented in AT (p=0.006; p<0.001 respectively) - No associations were found in COL11A1 rs3753841, COL11A rs1676486 and COL11A2 rs1799907 genotype or allele frequency distributions |
| Mokone et al. (2006) (43) | 72/129; AT subjects and healthy controls | AT 69.4%, CON 61.7% | AT 39.7 ± 15.3, CON 40.3 ± 11.0 | Achilles tendinopathy (not specified midportion or insertional) | - Frequency of distribution of COL5A1 BstUI RFLP A1, A2 and A3 alleles was significantly different (p=0.0009). The frequencies of the A1 and A3 alleles were higher in AT (A1 79.9%, A3 6.2%) and lower in AT for the A2 allele (13.9%). Odds ratio A2 allele negative 2.6 (95% CI 1.5-4.5, p=0.0005). - No differences were found in the distribution of the COL5A1 DpnII RFLP B1 and B2 alleles. - No differences were found in the genotype frequencies of the COL5A1 BstUI and DpnII RFLPs. |
| Nell et al. (2012) (44) | 166/358; AT subjects and healthy controls | NR | NR | Achilles tendinopathy (not specified midportion or insertional) | - CASP8 rs3834129 DD genotype (compared to DI, II and I genotype) was associated with AT (OR 1.67, 95% CI 1.08-2.60, p=0.0141) - CASP8 rs1045485 GC genotype (compared to GG, CC and C genotype) was associated with AT (OR 0.56, 95% CI 0.35-0.86, p=0.0094) - No associations were found in the genotype or allele frequency distributions for NOS3 rs1799983 and NOS2 rs2779249 |
| Posthumus et al. (2009) (45) | 85/126; AT subjects and healthy controls | AT 71.8%, CON 64.0% | AT 39.4 ± 14.7, CON 37.1 ± 10.6 | Achilles tendinopathy (not specified midportion or insertional) | - No associations were found in the genotype or allele frequency distributions of the COL1A1 rs1800012 Sp1-binding site polymorphism |
| Rahim et al. (2016) (46) | 195 (108 UK and 87 SA)/250 (130 UK and 120 SA); AT subjects and healthy controls | AT UK 58.6% and SA 70.5%, CON UK 63.1% and SA 63.3% | AT UK 45.2 ± 14.4 and SA 42.9 ± 13.6, CON UK 41.6 ± 11.6 and SA 37.3 ± 10.4 | Achilles tendinopathy (not specified midportion or insertional) | - VEGFA rs699947, rs1570360 and rs2010963 A-G-G inferred haplotype was overrepresented in AT (p=0.009) - No associations were found in the genotype or allele frequency distributions of VEGFA rs699947, VEGFA rs1570360, VEGFA rs2010963, KDR rs2071559 and KDR rs1870377 |
| Raleigh et al. (2009) (47) | 75/98; AT subjects and healthy controls | AT 73.0%, CON 67.0% | AT 40.5 ± 13.7, CON 36.8 ± 9.9 | Achilles tendinopathy (not specified midportion or insertional) | - MMP3 rs679620 GG genotype (compared to AG and AA genotype), SNP rs591058 CC genotype (compared to TC and TT genotype) and SNP rs650108 AA genotype (compared to GA and GG genotype) were associated with AT (OR 2.5, 95% CI 1.2-4.9, p=0.010; OR 2.3, 95% CI 1.1-4.5, p=0.023 and OR 4.9, 95% CI 1.0-24.1; p=0.043 respectively) - SNP rs679620 and rs591058 GC inferred haplotype, SNP rs679620, rs591058 and rs650108 ATG inferred haplotype and MMP3 rs679620 G allele and COL5A1 rs12722 T allele combination were overrepresented in AT (p=0.031, p=0.031 and p=0.006 respectively) - No associations were found in the genotype or allele frequency distributions of MMP3 rs591058 and rs650108 |
| Saunders et al. (2013) (52) | 179 (85 AUS and 94 SA)/339 (208 AUS and 131 SA); AT subjects and healthy controls | NR | NR | Achilles tendinopathy (not specified midportion or insertional) | - TNC rs1330363 G-allele was overrepresented in AT (p=0.020) - TNC rs2104772 T-allele was underrepresented in AT (p=0.017) - COL27A1 rs946053, TNC rs13321 and TNC rs2104772 CGA haplotype was over-represented in AT (p=0.019) - No associations were found in the genotype or allele frequency distributions of COL 27A1 rs4143245, rs1249744, rs753085 and rs946053 and TNC rs13321. No differences were found in the frequency of inferred haplotypes constructed from all seven variants |
| Saunders et al. (2014) (50) | 178 (84 AUS and 94 SA)/340 (207 AUS and 133 SA); AT subjects and healthy controls | AT AUS 61% and SA 67%, CON AUS 83% and SA 84% | AT AUS 40 ± 14 and SA 39 ± 15, CON AUS 39 ± 12 and SA 37 ± 11 | Achilles tendinopathy (not specified midportion or insertional) | - No associations were found in the genotype or allele frequency distributions of THBS2 rs9505888, rs6422747 and rs9283850 and COMP rs730070 and rs2849505 variants |
| Saunders et al. (2015) (51) | 179 (85 AUS and 94 SA)/339 (208 AUS and 131 SA); AT subjects and healthy controls | AT AUS 72.9% and SA 72.0%, CON AUS 40.2% and SA 63.2% | AT AUS 40.4 ± 14.2 and SA 39.7 ± 14.1, CON AUS 38.5 ± 12.4 and SA 37.3 ± 10.6 | Achilles tendinopathy (not specified midportion or insertional) | - COL5A3 rs1559186 GG genotype (compared to CC and CG genotype) was associated with AT (OR 0.452, 95% CI 0.198-0.940, p=0.017) - No associations were found in the genotype or allele frequency distributions of COL3A1 rs205615, rs3106796 and rs1800255, COL5A2 rs13031549 and rs4667264 and COL5A3 rs2303099 and rs2161468 |
| September et al. (2008) (54) | 93/131; AT subjects and healthy controls | AT 72.0%, CON 64.6% | AT 39.1 ± 14.3, CON 37.1 ± 10.4 | Achilles tendinopathy (not specified midportion or insertional) | - No associations were found in the genotype or allele frequency distributions of COL12A1 BsrI and AluI and COL14A1 BstYI and SspI - No differences were found in the frequency of inferred haplotypes constructed from COL12A1 BsrI and AluI and COL14A1 BstYI and SspI |
| September et al. (2009) (53) | 178 (85 AUS and 93 SA)/342 (210 AUS and 132 SA); AT subjects and healthy controls | AT AUS 72.9, CON AUS 40.2%. NR for SA | AT AUS 40.4 ± 14.2, CON AUS 38.5 ± 12.4. NR for SA | Achilles tendinopathy (not specified midportion or insertional) | - COL5A1 rs12722 CC genotype (compared to TC or TT genotype) was associated with AT in the AUS (OR 0.42, 95% CI 0.20-0.86, p=0.017) and SA population (OR 0.38, 95% CI 0.18-0.77, p=0.008) - COL5A1 rs3196378 AC genotype (compared to AA and CC) was associated with AT in the AUS population (OR 2.3; 95% CI 1.3-4.1, p=0.004) - COL5A1 rs12722 and rs3196378 T-C inferred haplotype was overrepresented in AT in the SA population (p<0.001) - No associations were found in the genotype or allele frequency distributions of COL5A1 rs11103544 and rs13946 in both groups, COL5A1 rs3196378 in the SA population and COL5A1 rs10858286, rs4504708 and rs3128575 in the AUS population |
| September et al. (2011) (77) | 175 (85 AUS and 90 SA)/369 (208 AUS and 161 SA); AT subjects and healthy controls | AT AUS 72.9% and SA 71.9%, CON AUS 39.6% and SA 64.4% | AT AUS 40.4 ± 14.2 and SA 39.8 ± 14.4, CON AUS 38.6 ± 12.4 and SA 36.3 ± 10.8 | Achilles tendinopathy (not specified midportion or insertional) | - Allele combination of the COL5A1 BstUI RFLP, IL-1beta-31T-->C, IL-1beta-511C-->T, IL-6-172G-->C and IL-1RNA VNTR polymorphisms was associated with AT (p=0.005) - No differences were found in the independent distributions of the genotype or allele frequencies at the IL-1beta-31T-->C, IL-1beta-511C-->T and IL-1RNA VNTR loci |
| **Medication** | | | | | |
| Van der Linden et al. (2002) (56) | 704/10 000; patients with an antibiotic drug prescription | NR | NR | Achilles tendinopathy (not specified midportion or insertional) | - Current use of fluoroquinolones ≥60 years was associated with AT (adjusted RR 3.1; 95% CI 2.0-4.8) - No associations were found for current, recent or past use of fluoroquinolones <60 years and for recent or past use of fluoroquinolones ≥60 years |
| AT, Achilles tendinopathy; AUS, Australian population; BMI, Body Mass Index; CI, Confidence interval; CON, Unaffected controls; HR, Hazard ratio; IL, Interleukin; INF, Interferon; km, kilometer; m, meter; ms; milliseconds; NR, Not reported; OR, Odds ratio; PC, Prospective cohort study; PDGF, Platelet-derived growth factor; RC, Retrospective cohort study; ROM, Range of motion; RR, Risk ratio; SA, South-African population; SD, Standard deviation; TNF, Tumor necrosis factor; VEGF; Vascular endothelial growth factor. | | | | | |
